# Health Effects of Electrolyzed Hydrogen Water for the Metabolic Syndrome and Pre-Metabolic Syndrome: A 3-Month Randomized Controlled Trial and Subsequent Analyses

**DOI:** 10.3390/antiox13020145

**Published:** 2024-01-24

**Authors:** Reiko Moribe, Marina Minami, Ryoji Hirota, Naw Awn J-P, Shigeru Kabayama, Masamitsu Eitoku, Keiko Yamasaki, Hajime Kuroiwa, Narufumi Suganuma

**Affiliations:** 1 Department of Environmental Medicine, Kochi Medical School, Kochi University, Nankoku 783-8505, Japan; reikojuriana@gmail.com (R.M.); ryoji.hirota@t.matsu.ac.jp (R.H.); jpnawawn@kochi-u.ac.jp (N.A.J.-P.); meitoku@kochi-u.ac.jp (M.E.); jm-keiko-yamasaki@kochi-u.ac.jp (K.Y.); nsuganuma@kochi-u.ac.jp (N.S.); 2 Integrated Center for Advanced Medical Technologies (ICAM-Tech), Kochi Medical School, Kochi University, Nankoku 783-8505, Japan; kuroiwa-hajime@kochi-u.ac.jp; 3 Graduate School of Health Science, Matsumoto University, Matsumoto 390-1295, Japan; 4 Nihon Trim Co., Ltd., 22F Herbis ENT Office Tower, 2-2-22, Umeda, Kita-ku, Osaka 530-0001, Japan; kabayama@nihon-trim.co.jp; 5 Graduate School of Science, Technology & Innovation, Kobe University, 1-1 Rokkoudai-cho Nada-ku, Kobe 657-8501, Japan

**Keywords:** electrolyzed hydrogen water, metabolic syndrome, waist circumference, physical activity, oxidative stress

## Abstract

We studied the effect of three months’ use of electrolyzed hydrogen water (EHW, Electrolyzed Hydrogen Water conditioner produced by Nihon Trim Co., Ltd.) on metabolic and pre-metabolic syndrome groups. This research was carried out jointly by Susaki City; Nihon Trim Co., Ltd.; and Kochi University as part of a local revitalization project with health as a keyword. A randomized, placebo-controlled, double-blind, parallel-group trial was conducted to evaluate the clinical impact of EHW on participants who suffered from metabolic syndrome or pre-metabolic syndrome. EHW was produced via electrolysis using a commercially available apparatus (Nihon Trim Co., Ltd., Osaka, Japan). During exercise, oxidative stress increases, and active oxygen species increase. In this study, we examined 181 subjects with metabolic syndrome or pre-metabolic syndrome. Among the group that drank EHW for 3 months, those who also engaged in a high level of physical activity showed a significant difference in waist circumference reduction. Although no significant difference was observed, several positive results were found in the participants who engaged in a high level of physical activity. Urinary 8-OHdG, urinary nitrotyrosine, HbA1c, and blood glucose levels increased in the filtered water (FW) group but decreased in the EHW group. High-sensitivity CRP increased less in the EHW group. 8-Isoprostane decreased more in the EHW group. In subgroup analysis, the EHW group showed a significantly greater reduction in waist circumference than the FW group only when controlled for high physical activity. Based on the result, we suggest that, among participants in the study who suffered from metabolic syndrome and pre-metabolic syndrome in which the level of active oxygen species is said to be higher than in healthy subjects, the group that consumed EHW and also engaged in a high level of physical activity experienced a suppressed or reduced increase in active oxygen species.

## 1. Introduction

Oxidative stress is defined as “an imbalance between oxidants and anti-oxidants in favor of the oxidants, leading to a disruption of redox signaling and control and/or molecular damage” [1]; it is implicated as a cause of age-related cognitive decline [2], neurodegenerative diseases [3,4], cardiovascular conditions [5,6], metabolic disorders [7,8], chronic inflammation, and cancers [9,10,11]. Thus, antioxidants, which scavenge or neutralize free radical or reactive oxygen species (ROS), have been investigated as a potential therapeutic intervention for various diseases. Recently, hydrogen, being recognized widely as having antioxidant properties, became attractive as a candidate in antioxidant research. Clinical studies demonstrated beneficial protective effects of hydrogen with no reported toxicity, for several health problems including chronic inflammatory condition [12], hypercholesterolemia and atherosclerosis [13], type 2 diabetes mellitus [14], metabolic syndrome [15,16], and Parkinson’s disease [17]. 

Metabolic syndrome is a condition in which visceral obesity leads to lipid abnormalities, high blood sugar, and high blood pressure. It is often caused by lack of exercise, overeating, etc. Improving one’s lifestyle can help prevent serious diseases in the future. In Japan, a person is diagnosed with metabolic syndrome if the waist circumference (abdominal circumference at the height of the navel) is 85 cm or more for men and 90 cm or more for women and if at least two out of three of blood pressure, blood sugar, and lipids are outside of standard values. In Japan, eight medical societies, including the Japanese Society of Internal Medicine, jointly formulated the diagnostic criteria for metabolic syndrome in 2005.

Obesity that does not meet the criteria for the diagnosis of metabolic syndrome but for which the risk can be reduced by weight loss is regarded as a “pre-metabolic syndrome group”, and it is necessary to promote lifestyle improvement to prevent the transition to metabolic syndrome. This categorization includes those whose abdominal circumference is above the standard but who have only one abnormality either of glucose metabolism, lipid metabolism, or blood pressure.

Based on animal studies, it was calculated that 1.8–3.6 mmol/day, which is equivalent to 3.6–7.2 mg/day of hydrogen administration, was required for biological effects in a 60 kg human [18]. Assuming the above value was derived from calculation using 10% of the lethal doses in animal studies and applying a conventional factor value of 10 to increase safety of the first human dose, the value for a human subject would become 0.36–0.72 mg/day of hydrogen [19]. Although hydrogen can be administered into our body via many routes, drinking electrolyzed hydrogen water (EHW) is the most feasible method. EHW can be obtained by directly dissolving hydrogen gas into water under high pressure [14], reacting metallic magnesium with water [15,16], or electrolyzing water to generate hydrogen [20,21]. However, the nanoscale hydrogen particles generated on the cathode side during water electrolysis [22] are likely to be different from those produced by simple infusion or reacting metals with water; this fact, perhaps, will bring out different effects in our biosystems.

Active oxygen species require a certain level of concentration for the maintenance of life, but when they are in excess they are thought to damage cells and trigger disease such as obesity, cancer, diabetes, and Alzheimer’s disease as well as aging. It has been pointed out that in the metabolic syndrome and the metabolic syndrome reserve groups, the blood oxide level is higher than that in a healthy subject.

In the latest research, it is becoming clear that drinking EHW reduces oxides in the blood and improves antioxidant activity, but the beneficial effect of long-term drinking of EHW in the metabolic syndrome reserve group is not yet clear.

In this study, EHW produced by Nihon Trim Electrolyzed Hydrogen Water Conditioner (hereinafter referred to as “test water conditioner”) was used over a long period to improve various blood test items of metabolic syndrome. We examined whether this was valid. Our research was carried out as a joint activity between Susaki City; Nihon Trim Co., Ltd.; and Kochi University as part of a “local revitalization project with health as a keyword”.

## 2. Materials and Methods

### 2.1. Study Design

A randomized, placebo-controlled, double-blind, parallel-group trial was conducted to evaluate the clinical impact of the EHW on the participants with metabolic syndrome and pre-metabolic syndrome, guided by the CONSORT 2010 Statement. This study initiated participant recruitment in July 2017 and concluded the follow-up in May 2019 in Susaki City, Kochi Prefecture, Japan. In our trial, we used REDCap [23], which is a widely used electronic data capture system, for block randomization, central allocation, and data collection. Participants were randomized via a computer-generated randomization scheme in a 1:1 ratio to study groups.

### 2.2. Study Population

The number of people with lifestyle-related diseases in Susaki is about 3300 (estimated value) in FY2015. Of these, approximately 1291 (National Health Insurance, 540, plus 751 others) are presumed to be in the metabolic syndrome and metabolic syndrome reserve group, who are the subjects of this study. One person from the same household can be the subject of this study.

**Figure 1:**
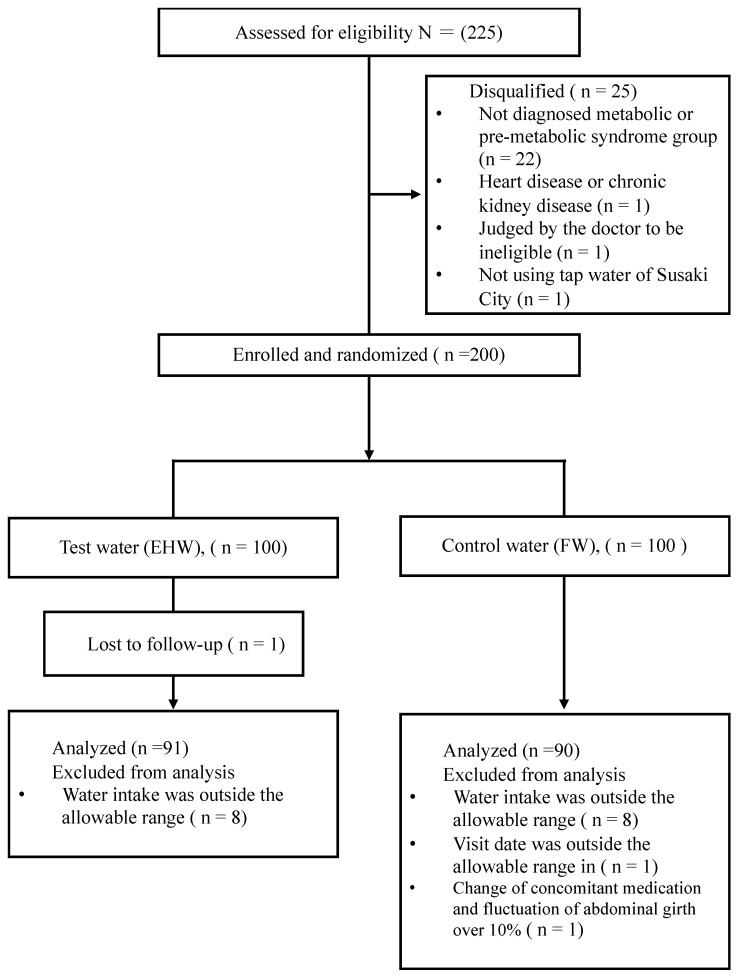
Flowchart for selection of participants. EHW, electrolyzed hydrogen water; FW, filtered water.

From this population, 225 Susaki Citizens aged ≥30 years who could install a test water conditioner were recruited at public spaces in Susaki City. After screening for eligibility at two hospitals in Susaki City, 25 of the 225 patients were disqualified because they did not meet inclusion criteria (Table 1) or met exclusion criteria (Table 2). Within these 25 cases, 22 cases were not diagnosed as a metabolic or pre-metabolic syndrome group, 1 case had heart disease or chronic kidney disease, 1 case was judged by the doctor to be ineligible for participation due to other reasons, and 1 case was judged to be ineligible for participation as the participant did not use the tap water of Susaki City. The 200 eligible cases were randomly allocated to EHW or FW groups. After randomization, one case in the EHW group was discontinued, there was a change in concomitant medication and fluctuation of waist circumference of over 10% in one case of the FW group, water intake was outside the allowable range in 16 cases (8 cases in the EHW group and 8 cases in the FW group), and the visit date was outside the allowable range in one case of the FW group. A total of 181 cases (91 cases in the EHW group and 90 cases in the FW group were analyzed (Figure 1).

### 2.3. EHW Preparation

Test water (EHW) and control water (filtered water: FW) were produced by using the improved devices of a commercially available apparatus: TRIM ION HYPER (Nihon Trim Co., Ltd., Osaka, Japan). The devices had identical outward appearances and method of operation. Hence, the subjects and the installers at the subjects’ houses were not able to distinguish the kinds of water. A total of 100 devices for EHW and 100 devices for FW were manufactured. An employee of the manufacture company Trim Electric Machinery Co., Ltd. (Kochi, Japan), allocated numbers 1–200 to A or B and also allocated A or B to EHW devices or FW devices using computer-aided random number generation. This information was kept in secret in order to blind all people and groups involved in the clinical trials. According to the data entry of subjects, the devices were sent to the subjects’ houses in numerical order. An employee of Nihon Trim Co., Ltd. (Osaka, Japan), installed the device on a tap at each subject’s house and explained how to use the device. Stainless bottles (350 mL) were handed out exclusively for use in drinking the test water, and subjects were asked to drink 350 mL of test water in the morning and afternoon and at night every day for 3 months. We confirmed via a questionnaire that the amount of water drunk was 1050 mL per day. We evaluated this record in detail and excluded from our analysis any individuals who were not drinking enough water.

### 2.4. Statistical Methods

The target sample size of the original study (n = 200 < each) was based on an estimated 10% difference between groups with a 1:1 ratio between them and calculated from the rationale of a statistical power of 90% and an alpha level of 0.05 using a two-sided log-rank test.

All values are expressed as the mean ± standard deviation (SD) or median (interquartile range) as appropriate. For comparisons between the two groups, Student’s *t*-test or the Mann-Whitney U test was used for continuous variables, and the chi-square test or Fisher’s exact test was used for the nominal variable, as appropriate. Values of *p* < 0.05 were considered statistically significant. Data were statistically analyzed using Stata/MP 13.1 software (Stata Corp., College Station, TX, USA).

After dividing physical activity levels into high, moderate, and low, a linear regression analysis was performed, adjusting for gender and smoking.

### 2.5. Study Method

The primary endpoint was a change in waist circumference from the baseline.

The secondary endpoints were amount of change from the baseline in metabolic syndrome evaluation items: body mass index (BMI), waist circumference, fasting blood glucose (FBS), glycosylated hemoglobin (HbA1c), total cholesterol, triglycerides (TGs), high-density lipoprotein cholesterol (HDL-C), low-density lipoprotein cholesterol (LDL-C), blood pressure, etc., and amount of change from baseline in items that affect metabolic syndrome: serum (high-sensitivity C-reactive protein (hs-CRP), interleukin 6 (IL6), adiponectin, monocyte chemoattractant protein-1 (MCP-1), tumor necrosis factor-α (TNF-α)) and in urine (8-hydroxy-2′-deoxyguanosine (8-OHdG), 8-isoprostane, nitrotyrosine).

BMI is a numerical value to judge whether weight is commensurate with height. The standard value for waist circumference is under 85 cm for men and under 90 cm for women. The standard value for systolic blood pressure is 129 mmHg or less. The standard value of diastolic blood pressure is 84 mmHg or less. Total cholesterol is determined using the screening test for primary and secondary hypercholesterolemia. HDL-C, which is also called good cholesterol and is reported to reduce risk for type 2 diabetes mellitus [24], is responsible for collecting excess cholesterol in the body, delivering it to the liver and eliminating it from the human body. The remaining cholesterol contained in lipoprotein particles, especially LDL-C, is called bad cholesterol, and a high value is a risk factor for coronary artery disease. TGs are important as an energy source, but excessive levels are a risk factor for arteriosclerosis. The FBS level is increased in diabetes. HbA1c, to which sugar is bound non-enzymatically, is indicated by the index of long-term glycemic control in the past 1–3 months in diabetic patients. Serum hs-CRP is a sensitive marker for inflammation and tissue damage. Urinary 8-OHdG is a biomarker indicating gene DNA damage caused by active oxygen. Isoprostane is a prostaglandin-like compound that is formed by free radical oxidation of phospholipids contained in cell membranes and lipoproteins and has been reported to be associated with smoking, diabetes, and arteriosclerosis. Nitrotyrosine is a marker of in vivo inflammation and nitric oxide production. IL-6 is a cytokine produced rapidly and transiently in response to infections and tissue injuries. Adiponectin acts to promote glucose uptake without insulin receptors and burning of fatty acids and acts to decrease intracellular fatty acids to increase sensitivity of insulin receptors, which is inversely correlated with visceral fat. MCP-1 is thought to be involved in the infiltration of monocytes and T cells in various inflammatory diseases such as arteriosclerosis, delayed allergy, rheumatoid arthritis, and lung disease. TNF-α is an inflammatory cytokine produced by macrophages. 

Metabolic syndrome evaluation items (anthropometry, glucose metabolism, lipid, and blood pressure) and hs-CRP were measured at two hospitals in Susaki City at baseline and 3 months later, and changes were calculated. Serum and urine samples collected in these hospitals at these timepoints were used for the enzyme-linked immunosorbent assay (ELISA) measurements of serum markers (IL6, adiponectin, MCP-1, TNF-α; BioLegend, San Diego, CA, USA), 8-OHdG (Nikken Seil, Shizuoka, Japan), 8-isoprostane (Oxford Biomedical Research, Oxford, MI, USA), and nitrotyrosine (StressMarq Biosciences, Victoria, BC, USA).

The information of physical activity levels and smoking was collected with a self-administered questionnaire. Physical activity was evaluated with the International Physical Activity Questionnaire—Short Form (IPAQ-SF) [25]. The IPAQ-SF consists of 7 items, which estimate the time spent for the four types of physical activity (vigorous-intensity activity, moderate-intensity activity, walking, and sitting) in the past 7 days. The time spent in the four types of physical activity was multiplied by their estimated intensity in metabolic equivalents (METs) and summed to obtain an overall estimate of weekly physical activity. MET intensities were vigorous (8 METs), moderate (4 METs), and walking (3.3 METs). Three physical activity levels (low, moderate, and high) were defined based on an overall estimate of physical activity. High activity achieved a minimum of 3000 MET-min/week. Moderate activity was moderate or vigorous activity achieving at least 600 MET-min/week. Low activity indicated individuals who did not meet criteria for the moderate or vigorous categories (<599 MET-min/week).

### 2.6. Ethical Considerations

This study protocol was reviewed and approved by the Ethics Committee of Kochi Medical School (approved 25 September 2017, approval number 29-110). The clinical trials protocol was registered on 28 September 2017, UMIN000029321. 

## 3. Results

The baseline data show significant differences in waist circumference, total cholesterol, and TGs (Table 3). We found a significant difference (*p*-value = 0.00) in waist circumference between groups. The waist circumference of the EHW group (N = 91) was 94.02 cm, but that of the FW group (N = 90) was 97.79 cm. The waist circumference of the EHW group was smaller than that of the FW group. We found significant differences (*p*-value = 0.00) in total cholesterol between groups. The total cholesterol of the EHW group (N = 91) was 217.44, but that of the FW group (N = 90) was 202.53. The total cholesterol of the EHW group was higher than the FW group. We found significant differences (*p*-value = 0.00) in the TGs between the groups. The value for the EHW group (N = 91) was 182.66, but that of the FW group (N = 90) was 143.24. The TG levels of the EHW group were greater than those of the FW group. 

The values in this report show the amount of change from Visit 1 (baseline) to Visit 4 (3 months after participants started drinking). When the value of the amount of change is positive, the measured value of Visit 4 is higher than that of Visit 1, and when the value of the change is negative, the measured value of Visit 4 is lower than that of Visit 1. 

### 3.1. Main Analysis

181 cases (91 cases in the EHW group and 90 cases in the FW group) were assessed with intention-to-treat analysis as randomly assigned (Table 4). The main analysis shows no significant difference in either the primary endpoint (change in waist circumstance) or secondary endpoint.

### 3.2. Stratified Analysis

Because no significant difference was found in the main analysis, we employed stratified analysis. In each stratified analysis, 181 cases were analyzed as in the main analysis. When results were stratified by sex, no significant difference was found in either men or women. When results were stratified by the presence of a history of hypertension, no significant difference was found in either the group with hypertension or the group without hypertension. When results were stratified by the presence of diabetes, we found a significant difference (*p*-value 0.03) in the blood glucose levels in the group without diabetes. The EHW group (N = 78) showed a decrease in blood glucose levels (2.32), but the FW group (N = 66) increased by 0.53. No significant difference was found in the group with diabetes. When results were stratified by the presence of dyslipidemia, no significant difference was found in either the group with dyslipidemia or the group without dyslipidemia.

Similar stratified analysis was applied to health behaviors. When results were stratified by drinking (alcohol) habit, we found a significant difference (*p*-value 0.04) in blood glucose levels in the group without a drinking habit. The EHW group (N = 37) showed an increase in blood glucose levels (0.76), but the FW group showed a decrease (6.20). We found a significant difference (*p*-value 0.04) in the change in body weight in the group with a drinking habit. The EHW group (N = 54) decreased by 0.79 in body weight, but the FW group decreased by 0.03. When results were stratified by smoking status, no significant difference was found in either group with the Brinkman index more than 700 nor with the group with the Brinkman index less than 700.

With high physical activity, we found a significant difference (*p*-value 0.01) in the amount of change in waist circumference in the groups (Table 5) The EHW group (N = 15) decreased by 1.87 cm, but the FW group (N = 12) increased by 0.96 cm.

With moderate physical activity, we found significant differences (*p*-value 0.02) in the amount of change in 8-OhdG (Table 6). The EHW group (N = 24) increased by 0.79, but the FW group (N = 23) decreased by 0.73.

With low physical activity, we found a significant difference (*p*-value 0.02) in the amount of change in total cholesterol (Table 7). The EHW group (N = 45) decreased by 9.84, but the FW group (N = 44) increased by 1.00. Also, we found a significant difference (*p*-value 0.02) in the amount of change in HDL-C. The EHW group (N = 45) decreased by 1.49, but the FW group (N = 44) decreased by 2.18.

Table 8 presents the association between waist circumference and EHW for physical activity level. Based on the “Guidelines for Data Processing and Analysis of the International Physical Activity Questionnaire (IPAQ)—Short and Long Forms—” in 165 cases for which questionnaires were completed, the participants were stratified into three groups: high physical activity, intermediate physical activity, and low physical activity. After dividing physical activity levels into high, moderate, and low, and after adjusting for gender and smoking, there was a significant association of −2.72 (95% confidence interval: −5.04, −0.41) for the high-physical-activity group.

No adverse events were observed among any of the groups in this study.

## 4. Discussion

Although no significant difference was observed in the randomized control trial, positive results were found in the sub-group analysis of the high-physical-activity population. The EHW successfully reduced waist circumference by 1.78 cm after 3 months’ intervention, in the high-physical-activity group. Such an effect was not affected by adjusting other factor like smoking. The Ministry of Health, Labour and Welfare of Japan describes in the guidance book for the people with metabolic syndrome and pre-metabolic syndrome that a reduction of 1 cm in waist circumference is equivalent to a reduction of 1 kg in visceral fat [26]. Karlsson et al. reported that an increase of 1 kg in visceral adipose increases the risk of type 2 diabetes with an odds ratio of 7.34 (95% CI = 4.48–12.0) in females and an odds ratio of 2.50 (95% CI = 1.98–3.14) in males by analyzing the developed sex-stratified, nonlinear prediction models for visceral adipose tissue mass using the UK Biobank cohort (n = 325,153) [27]. From this information, it is estimated that the risk of type 2 diabetes at least reduced to 1/7.34 in females and 1/2.50 in males of the subjects with 3 months’ intervention of EHW in the high-physical-activity group. As for the reduction in body weight by 0.2 kg on average, it is speculated that the reduction is the result of a reduction in visceral adipose and an increase in muscle. 

In addition to the baseline oxidatively stressed status of metabolic and pre-metabolic syndrome participants [28], the higher-physical-activity group should have considerable oxidative damage, as the major source of oxidative stress is from energy production by mitochondria [29]. This suggests the presence of a possible redox mechanism that consequently affects physique, especially among the group of highly physically active subjects [30]. The differential results in waist circumference change when stratifying by the intensity of physical activity are understandable, as the reduction in waist circumference is proven to be achieved by prescribed unsupervised exercise [31]. When exercise is combined well with diet control, it can totally cure metabolic syndrome after 3 weeks’ intervention [32]. Thus, high physical activity played a key role in reduction in waist circumference, which is among the criteria of metabolic syndrome. The abovementioned possibly increased oxidative damage caused by intense physical activity should be treated by scavengers, otherwise it will cause adverse outcomes [29].

The EHW intake should have effectively obscured the increased oxidative stress through intense physical activity. The difference between intervention and control groups among the high-physical-activity strata was significant enough to be detected even with the small sample size of the sub-group analysis. Findings that showed the tendency of the EHW effect to reduce oxidatively damaged biomolecules include the results for urinary 8-OHdG and nitrotyrosine. Such a tendency was also observed for HbA1c and blood glucose levels that increased in the FW group, whereas they decreased in the EHW group. In addition, the anti-inflammation effect of EHW is indicated by the change in hs-CRP, adiponectin, MCP-1, and TNF-α, which were less increased or decreased in the EHW group. Urinary 8-isoprostane tended to decrease more in the EHW group. This tendency of effect of EHW is in accordance with previous research that showed decreased superoxide dismutase after EHW consumption [25,26]. 

These findings may explain the reduced waist circumference among the high-physical-activity group. In the subgroup of high physical activity, the FW intake group had an increase in oxidative damage, while the EHW intake group showed a decrease or less of an increase in oxidative damage. It was suggested that drinking EHW suppressed the increase in oxidative damage, which should be caused by high physical activity. Consequently, it may have contributed to the metabolism system and waist circumference reduction. Further studies with larger sample sizes are needed to confirm the combined effect of EHW with high physical activity. 

This study also had several limitations. First, some of the baseline characteristics for the EHW group and FW group were significantly different, even after the randomization. Insignificant results of this RCT may be, at least partially, derived from the apparent uneven distribution of baseline characteristics, including waist circumference, which is the primary endpoint; TGs, which are among the secondary endpoints; and TNF-α. Thus, performing subsequent stratified and sub-group analyses was important to investigating overlooked associations. Second, our study was conducted over a three-month period, which might be too brief to detect the effect of EHW consumption. LeBaron et al. reported the results of a similar 24-week intervention study, the results of which showed that supplementation with high-concentration H_2_-rich water significantly reduced blood cholesterol and glucose levels, attenuated serum HbA1c, and improved biomarkers of inflammation and redox homeostasis as compared to a placebo (*p* < 0.05) [33]. Furthermore, H_2_ tended to promote a mild reduction in BMI and waist-to-hip ratio in their report [34]. We may need to increase the timespan of the study to confirm positive results of the effects of EHW. Third, the sample size might not have been sufficient to detect any difference in the main study. Fourth, because the total amount of water was not controlled, the effect of drinking water drawn from sources other than equipment supplying EHW and FW is unknown. It should be noted that the results on the EHW effect in the high-physical-activity group are also only a proof of concept and are insufficient as a basis for clinical recommendation. Finally, new findings of the EHW effect in the high-physical-activity group are based on data collected using self-administered questionnaires. Therefore, the possibility of misclassification of the physical activity group, which may occur randomly in both the EHW and FW groups, should be noted.

## 5. Conclusions

In this study, as a health effect of EHW for metabolic syndrome and pre-metabolic syndrome, a decrease in waist circumference was observed only in the high-physical-activity population. We suggest that, for those suffering from metabolic syndrome and pre-metabolic syndrome, in which the level of oxidative damage is said to be higher than in healthy subjects, high physical activity will help to reduce waist circumference. Physical activity or exercise will increase oxidative damage, but the EHW intake will suppress or lessen these effects.

## Figures and Tables

**Table 1:** Inclusion criteria.

I. Age 30 years or older
II. Those who have been diagnosed with any of the following (1) Metabolic syndrome group Waist circumference (85 cm or more for men, 90 cm or more for women) plus 2 or more of the following (a) Glucose metabolism (FBS 110 mg/dL or more) (b) Lipids (TGs 150 mg/dL or more or HDL-C 40 mg/dL or less) (c) Blood pressure (systolic 130 mmHg or higher and/or diastolic 85 mmHg or higher) (2) Pre-metabolic syndrome group (d) Waist circumference (85 cm or more for men, 90 cm or more for women) Those with abnormalities either of glucose metabolism, lipids, or blood pressure (e) Waist circumference is below the standard value, but BMI is 25 or more and one or more of the above risks are present

BMI, body mass index; FBS, fasting blood glucose; HDL-C, high-density lipoprotein cholesterol; TGs, triglycerides. Presence of drug treatment for hypertriglyceridemia, hypo-HDL-C, hypertension, and diabetes is considered to have applicable abnormalities.

**Table 2:** Exclusion criteria.

I. Those who are judged by doctors to be ineligible to participate (1) Those with heart disease or chronic kidney disease (2) Those with a disease associated with weight loss (hyperthyroidism, etc.) (3) Person with a serious disease or malignant tumor * Malignant tumors can be incorporated if they have been cured for 5 years (4) Those judged by the doctor to be ineligible for participation due to other reasons II. Person who has had an EHW device installed before participating in the study III. Those from whom consent has not been obtained

EHW, electrolyzed hydrogen water.

**Table 3:** Baseline characteristics of EHW group and FW group.

Total (N = 181)	Baseline Mean (SD)				
	EHW (N = 91)		FW (N = 90)		*p*-Value
Age	57.74	(11.64)	59.26	(12.06)	0.39
Sex, n (%)					0.19
Male	64	(70.3)	55	(61.1)	
Female	27	(29.7)	35	(38.9)	
Smoking, n (%)					0.60
Smoker	15	(16.5)	18	(20.0)	
Former Smoker	31	(34.1)	24	(26.7)	
Non-Smoker	40	(44.0)	40	(44.4)	
Missing	5	(5.5)	8	(8.9)	
Physical activity, n (%)					0.96
Low	45	(49.5)	44	(48.9)	
Moderate	24	(26.4)	23	(25.6)	
High	15	(16.5)	14	(15.6)	
Missing	7	(7.7)	9	(10.0)	
Height	164.60	(8.94)	163.04	(9.77)	0.26
Body weight	74.45	(10.79)	75.80	(13.66)	0.46
BMI	27.44	(3.00)	28.40	(3.68)	0.06
Waist circumference	94.02	(7.16)	97.79	(9.06)	0.00 *
Systolic blood pressure	141.26	(17.78)	143.02	(17.55)	0.50
Diastolic blood pressure	85.11	(11.66)	84.76	(12.18)	0.84
Total cholesterol	217.44	(35.54)	202.53	(30.02)	0.00 *
HDL-C	59.80	(15.48)	58.03	(16.25)	0.45
LDL-C	127.96	(33.08)	121.59	(26.91)	0.16
TGs	182.66	(110.54)	143.24	(66.77)	0.00 *
FBS	111.07	(17.17)	115.72	(27.14)	0.17
HbA1c	5.94	(0.67)	6.10	(0.92)	0.17
hs-CRP	0.13	(0.22)	0.12	(0.14)	0.80
8-OHdG	2.61	(2.93)	2.94	(3.34)	0.49
8-Isoprostane	3.09	(3.71)	2.82	(2.35)	0.55
Nitrotyrosine	22,027.68	(23,154.13)	23,644.99	(29,587.66)	0.68
IL-6	17.38	(109.48)	4.99	(10.08)	0.29
Adiponectin	18.49	(11.03)	19.94	(15.41)	0.47
MCP-1	184.77	(89.13)	173.59	(90.32)	0.40
TNF-α	2.37	(8.75)	0.49	(2.09)	0.05

BMI, body mass index; EHW, electrolyzed hydrogen water; FBS, fasting blood glucose; FW, filtered water; HbA1c, glycosylated hemoglobin, HDL-C, high-density lipoprotein cholesterol; hs-CRP, high-sensitivity C-reactive protein; IL-6, interleukin 6; LDL-C, low-density lipoprotein cholesterol; MCP-1, monocyte chemoattractant protein-1; 8-OHdG, 8-hydroxy-2′-deoxyguanosine; SD, standard deviation; TGs, triglycerides; TNF-α, tumor necrosis factor-α. * Indicates significant results in the Student’s *t*-test.

**Table 4:** Change from baseline after 3 months for EHW group and FW group.

Total (N = 181)	EHW (N = 91)		FW (N = 90)		*p*-Value
	Mean (SD)				
Body weight	−0.29	(1.61)	−0.10	(2.18)	0.51
BMI	−0.10	(0.58)	−0.06	(0.79)	0.65
Waist circumference	−1.27	(3.48)	−1.05	(3.15)	0.65
Systolic blood pressure	−5.96	(16.56)	−6.62	(15.60)	0.78
Diastolic blood pressure	−3.84	(10.10)	−3.94	(11.02)	0.94
Total cholesterol	−7.18	(29.24)	−1.22	(20.56)	0.12
HDL-C	−0.31	(6.76)	0.98	(6.97)	0.21
LDL-C	−3.55	(24.06)	−2.06	(17.97)	0.64
TGs	−13.52	(72.13)	−4.99	(63.80)	0.40
FBS	−1.18	(15.91)	−1.71	(13.32)	0.81
HbA1c	−0.03	(0.22)	−0.03	(0.35)	0.95
hs-CRP	−0.02	(0.26)	0.00	(0.18)	0.50
8-OHdG	−0.31	(2.76)	−0.35	(0.26)	0.91
8-Isoprostane	−0.43	(3.39)	−0.20	(1.82)	0.58
Nitrotyrosine	−2960.40	(21,224.07)	−3124.72	(33,317.66)	0.97
IL-6	−5.86	(48.54)	−1.41	(11.20)	0.40
Adiponectin	0.07	(5.61)	1.03	(6.82)	0.30
MCP-1	0.00	(84.57)	3.67	(62.09)	0.74
TNF-α	4.92	(20.91)	6.62	(27.68)	0.64

Abbreviations are the same as in Table 3.

**Table 5:** Change from baseline after 3 months for EHW group and FW group with high physical activity.

Total (N = 29)	High Physical Activity Mean (SD)				
	EHW (N = 15)		FW (N = 14)		*p*-Value
Body weight	−0.20	(1.67)	−0.08	(1.30)	0.83
BMI	−0.08	(0.59)	−0.03	(0.50)	0.82
Waist circumference	−1.87	(2.38)	0.96	(3.32)	0.01 *
Systolic blood pressure	−6.00	(12.50)	−6.07	(9.24)	0.99
Diastolic blood pressure	−5.73	(10.10)	−2.00	(9.10)	0.31
Total cholesterol	−1.13	(17.79)	−3.43	(14.54)	0.71
HDL-C	1.87	(5.91)	−0.36	(3.82)	0.24
LDL-C	−0.27	(15.38)	−1.64	(16.00)	0.82
TGs	−18.60	(75.90)	−20.07	(34.54)	0.95
FBS	−2.47	(15.93)	1.64	(9.90)	0.42
HbA1c	−0.08	(0.30)	0.09	(0.17)	0.07
hs-CRP	0.01	(0.09)	0.02	(0.05)	0.72
8-OHdG	−1.33	(3.61)	0.03	(2.38)	0.25
8-Isoprostane	−0.92	(1.22)	−0.44	(0.98)	0.25
Nitrotyrosine	−12,150.80	(20,505.49)	2693.93	(26,684.15)	0.10
IL-6	1.61	(14.77)	2.61	(10.64)	0.84
Adiponectin	−0.12	(4.59)	0.99	(5.83)	0.57
MCP-1	−1.95	(49.48)	−19.61	(44.68)	0.32
TNF-α	5.63	(19.43)	6.57	(14.00)	0.88

Abbreviations are the same as in Table 3. * Indicates significant results in the Student’s *t*-test.

**Table 6:** Change from baseline after 3 months for EHW group and FW group with moderate physical activity.

Total (N = 47)	Moderate Physical Activity Mean (SD)				
	EHW (N = 24)		FW (N = 23)		*p*-Value
Body weight	−0.07	(1.55)	−0.56	(1.89)	0.33
BMI	−0.03	(0.56)	−0.19	(0.70)	0.38
Waist circumference	−1.66	(3.65)	−1.83	(2.95)	0.86
Systolic blood pressure	−6.62	(15.65)	−10.17	(15.63)	0.44
Diastolic blood pressure	−5.62	(10.88)	−6.57	(13.92)	0.80
Total cholesterol	3.79	(28.55)	−3.09	(31.31)	0.44
HDL-C	0.54	(6.85)	1.22	(8.46)	0.76
LDL-C	2.46	(24.69)	−3.91	(25.33)	0.39
TGs	−14.08	(77.42)	−24.22	(55.97)	0.61
FBS	−1.88	(6.54)	−3.83	(15.25)	0.57
HbA1c	−0.02	(0.16)	−0.07	(0.57)	0.69
hs-CRP	−0.01	(0.06)	−0.01	(0.12)	0.96
8-OHdG	0.79	(1.97)	−0.73	(2.17)	0.02 *
8-Isoprostane	−0.71	(6.27)	0.26	(2.23)	0.49
Nitrotyrosine	1065.71	(19,083.42)	−1639.26	(27,737.54)	0.70
IL-6	−0.88	(7.08)	−2.17	(8.05)	0.56
Adiponectin	1.23	(5.52)	1.66	(6.42)	0.81
MCP-1	−9.47	(55.77)	7.82	(39.66)	0.23
TNF-α	5.07	(13.95)	13.71	(50.31)	0.42

Abbreviations are the same as in Table 3. * Indicates significant results in the Student’s *t*-test.

**Table 7:** Change from baseline after 3 months for EHW group and FW group with low physical activity.

Total (N = 89)	Low Physical Activity Mean (SD)				
	EHW (N = 45)		FW (N = 44)		*p*-Value
Body weight	−0.45	(1.68)	0.09	(2.52)	0.24
BMI	−0.15	(0.61)	−0.00	(0.91)	0.36
Waist circumference	−0.98	(3.76)	−1.03	(2.93)	0.94
Systolic blood pressure	−6.40	(18.53)	−3.57	(16.77)	0.45
Diastolic blood pressure	−3.00	(10.18)	−3.55	(10.09)	0.80
Total cholesterol	−9.84	(24.68)	1.00	(16.36)	0.02 *
HDL-C	−1.49	(6.98)	2.18	(6.98)	0.02 *
LDL-C	−2.98	(18.34)	−1.70	(15.03)	0.72
TGs	−9.31	(66.42)	7.66	(74.03)	0.26
FBS	0.04	(20.18)	−2.43	(13.96)	0.50
HbA1c	−0.04	(0.20)	−0.06	(0.25)	0.69
hs-CRP	−0.02	(0.33)	−0.02	(0.15)	1
8-OHdG	−0.58	(2.78)	−0.48	(3.01)	0.87
8-Isoprostane	−0.15	(1.35)	−0.29	(1.85)	0.68
Nitrotyrosine	−2500.82	(23,135.81)	−1682.43	(32,939.98)	0.89
IL-6	−0.77	(7.44)	−1.46	(9.10)	0.70
Adiponectin	−0.63	(6.17)	1.07	(7.88)	0.26
MCP-1	−9.18	(69.36)	−3.22	(52.64)	0.65
TNF-α	2.84	(20.55)	3.80	(13.34)	0.80

Abbreviations are the same as in Table 3. * Indicates significant results in the Student’s *t*-test.

**Table 8:** Waist circumference change from baseline after the 3-month EHW intervention stratified by intensity of physical activity.

	Low	Moderate	High
	*β* (95% CI)	*β* (95% CI)	*β* (95% CI)
EHW	0.05 (−1.39, 1.49)	0.03 (−1.97, 2.04)	−2.72 (−5.04, −0.41) *
Gender	−0.42 (−2.03, 1.2)	0.34 (−1.97, 2.66)	1.69 (−0.67, 4.04)
Smoking	−0.16 (−1.17, 0.85)	0.49 (−0.84, 1.83)	−1.07 (−2.77, 0.63)

CI, confidence interval; EHW, electrolyzed hydrogen water. * Indicates significant results in the adjusted regression analysis.

## Data Availability

The data presented in this study are available on request from the corresponding author.
